# Trainability of repetitions-in-reserve estimation in younger and older adults: a parallel-group repeated-measures study

**DOI:** 10.1186/s13102-026-01997-y

**Published:** 2026-08-20

**Authors:** Tim Wiedenmann, Daniel Kunellis, Ludwig Rappelt, Steffen Held, Pamela Wicker, Lars Donath

**Affiliations:** 1 Department of Applied Exercise Science, German Sport University Cologne, Cologne, Germany; 2 Department of Movement and Training Science, University of Wuppertal, Wuppertal, Germany; 3 Department of Management & Sport, IST-University of Applied Sciences, Dusseldorf, Germany; 4 Department of Sports Science, Bielefeld University, Bielefeld, Germany

**Keywords:** Autoregulation, Strength training, Resistance training, Monitoring, Training prescription, Aged

## Abstract

**Background:**

Effort-based resistance training (RT) approaches, such as *repetitions (reps) in reserve* (RIR), aim to enhance RT individualization, which is of particular relevance for older adults. However, evidence regarding the accuracy of RIR estimation and its trainability in this population remains limited. This study aimed to (I) compare RIR estimation accuracy between younger and older adults, and (II) determine whether six training sessions improve estimation accuracy in both groups.

**Methods:**

The study followed a parallel-group repeated-measures-design. Thirteen younger adults (33 ± 7 years; 9 females, 4 males) and thirteen older adults (67 ± 4 years; 7 females, 6 males), completed six sessions across six weeks. Each session comprised three sets of bench press and leg press at randomized loads (75%-85% one rep maximum). During each set, participants verbally indicated the rep at which they estimated having 4 and 2 RIR (4RIR, 2RIR) and then continued the set to concentric muscle failure.

**Results:**

Linear mixed-effects models showed a significant main effect of training session on mean absolute error for both exercises at 2RIR and 4RIR, with MAE decreasing by up to 2.3 reps from Session 1 to Session 6, and no significant effects of age group or session x group interactions.

**Conclusions:**

The present findings suggest that RIR estimation accuracy is a trainable skill for both younger and older adults, with no evidence of age-related differences in baseline accuracy or trainability. These findings extend the limited evidence on RIR-based autoregulation to older adults and suggest that, after brief familiarization, RIR-based resistance training may represent a feasible strategy to support individualized exercise prescription in older adults.

**Supplementary Information:**

The online version contains supplementary material available at 10.1186/s13102-026-01997-y.

## Background

Resistance training (RT) is considered essential for older adults to support health and independent living by preserving muscle mass, strength, and physical function [1]. The level of RT effort, which can be defined as the proximity to concentric muscle failure at the end of an exercise set [2], has implications for fatigue management and may influence the magnitude of hypertrophy, particularly when mechanical load is lower [3–5]. Researchers and practitioners commonly operationalize RT effort using the repetitions (reps) in reserve (RIR) approach, which represents the number of additional reps that could have been performed after the end of a set, if it had been continued to concentric muscle failure [6].

Other key RT variables such as intra-set volume (i.e., the number of reps performed within a set) and training intensity, most commonly defined as the mechanical load relative to a maximal load (e.g., one-rep maximum; 1RM) [7, 8], are inherently interrelated with RIR. This relationship enables RIR to function as a flexible autoregulatory framework, as a given RIR target adapts the performed volume to an individual’s momentary performance capacity, even when the load is held constant. Consequently, the RIR method represents a versatile approach to enhance the individualization and safety of RT by accounting for intra- and inter-individual variability in training prescription, monitoring, and regulation [6, 9].

As an autoregulatory RT prescription method, the RIR approach relies on individuals’ subjective estimation of their true proximity to muscle failure within a set. The accuracy of this estimation has been demonstrated to be context-specific. Specifically, accuracy appears to be superior when sets are performed closer to muscle failure or with higher loads, which typically result in fewer total reps being completed [9–11]. Although several reviews and meta-analyses have highlighted the importance of familiarity with the RIR method for accurate estimation, studies explicitly investigating familiarization or training effects remain scarce and available findings are inconsistent [6, 9, 12]. Two studies examining the training effects on RIR estimation accuracy during barbell bench press (BP) reported no meaningful improvements following one session or six weeks of training [13, 14]. In contrast, other studies reported small improvements in accuracy after five weeks of training for BP and squat, and after a single familiarization session for leg press (LP) but not for chest press [15, 16].

Notably, the rather small body of evidence on RIR estimation accuracy is derived predominantly from younger, resistance-trained individuals [11]. This is despite recent reviews and meta-analyses [9, 12] consistently emphasizing the potential advantages of RIR usage in special populations, such as older adults, who typically exhibit greater intra- and inter-variability in muscular strength and muscle mass [12, 17]. In older adults in particular, inaccurate RIR estimation may lead to unintended overreaching or underloading, with potential consequences for training efficacy. Findings from a first study specifically examining older adults indicate that individuals may inaccurately estimate RIR [18], highlighting the need for further targeted investigations into both the accuracy and its trainability in this population.

Hence, the objectives of this study were to (I) investigate RIR estimation accuracy during BP and LP in younger and older adults, and (II) determine whether six consecutive training sessions can lead to meaningful improvements in RIR estimation accuracy in both age groups. By addressing these objectives, the study aims to provide novel evidence that may inform future familiarization strategies for the practical application of RIR-based training prescription, regulation, and monitoring in older adults.

## Methods

### Study design

The study followed a parallel group-, repeated-measures-design. Over the course of seven weeks participants completed a total of seven sessions. The first session consisted of a familiarization with the concept of training to concentric muscle failure and the communication cues required for the following sessions. The following six sessions consisted of a BP and LP training in which participants were instructed to estimate the exact rep after which they were able to perform four (4RIR) or two (2RIR) more reps, respectively. To quantify estimation error, participants performed each set to concentric muscle failure. A 1RM assessment for the BP and LP exercise was conducted before each of the six training sessions. The study protocol complied with the Declaration of Helsinki, fulfilled the international ethical standards [19] and was approved by the Local Ethical Committee of the University of Wuppertal (SK/Co 250708).

### Participants

The required sample size was determined a-priori using G*Power (Version 3.1.9.6). The analysis was conducted for an “ANOVA: Repeated measures, within factors”, assuming a medium main effect of time (Cohen’s f = 0.25), a moderate correlation between repeated measures of 0.5, a conservative non-sphericity correction factor ε of 0.75, a significance level of α = 0.05, and a desired statistical power of 0.80. This choice represents a conservative estimate relative to reported within-group changes in RIR accuracy, reported in previous research [16]. The calculation indicated a required total sample size of at least 24 participants (12 per group). This calculation targeted the within-subjects main effect of session and not the between-group differences or group x session interactions. These differences should therefore be considered exploratory. Accounting for a moderate dropout rate, the targeted sample size was 30 participants. Hence, 30 healthy recreationally active young (age: 18–40) and older adults (age: 60–80), with prior experience in RT but without experience in RIR and RPE training regulation, were initially enrolled in this study and allocated to their respective age groups (*younger adults; older adults*). Four participants (two from each group) withdrew from the study due to scheduling conflicts (*n* = 2), prolonged illness (*n* = 1) and back pain not related to the training (*n* = 1). Consequently, a total of 26 participants (*younger adults:n* = 13; female (f) = 9; male (m) = 4; *older adults:n* = 13; f = 7; m = 6) completed the study. No relevant health impairments were reported by any of the participants at the time of the investigation. All participants signed an informed written consent after receiving comprehensive study information.

### Procedures

The seven sessions were separated by five to ten days and lasted less than 60 min each. Every session was supervised by one of the researchers and conducted individually for each participant.

After the initial familiarization session the following six sessions were all conducted identically. Sessions started with the LP followed by the BP. Before the training sets the respective 1RM was assessed. According to standard procedures [20], participants warmed up on a cycling ergometer or cross trainer (5 min; low intensity) followed by three submaximal sets of the respective exercise with increasing load (approx. 40–50%1RM; 60–70%; 80–90%1RM) and decreasing number of reps (10; 5; 2).

The BP was performed using a barbell (10 kg; 2 kg if required) loaded with additional weight plates. The participants positioned themselves on a flat bench and were instructed to keep their upper back, glutes, and head in contact with the bench and the feet in contact with the floor at all times. The exercise was performed with a complete lockout (full extension of the elbows) at the top end of the movement. The barbell had to touch the chest at the lower end of the movement.

The LP was performed using a horizontal, sled-based LP machine with the resting position at the bottom of the movement. The machine was adjusted individually so that an imaginary line from the top of the knee to the hip crease was perpendicular to the ground. This position was defined as the starting position and lower end of the movement. The top end of the movement was defined as a knee angle slightly below full extension, thereby avoiding hyperextension.

After the 1RM assessment participants performed three sets of the respective exercises. The 1RM assessment and the training sets were separated by a self-selected rest period with a minimum duration of five minutes. The exercises were performed with a random distinct load corresponding to 75% and 85% of the tested 1RM for each individual set and session. This procedure was employed to ensure that improvements in estimation accuracy could be attributed to a true training effect rather than the participants simply counting an exact number of reps and recalling the results for the next set or session. Each of the sets were separated by a self-selected rest period with a minimum duration of five minutes. During each set, participants were instructed to verbally cue, in real time, the exact rep after which they estimated being able to perform four (4RIR) or two (2RIR) more reps. To ensure independent estimation for the two RIR targets, the participants were specifically instructed to evaluate each of the RIR targets separately. Afterwards every set was continued until concentric muscle failure. Concentric muscle failure was defined as either *repetition maximum* or *momentary failure* as proposed by Steele and colleagues (2017) [2].

Participants were informed about the purpose of the training sessions but received no additional explicit feedback regarding their performance or estimation error both within session and after session cessation. Completed number of reps at concentric muscle failure, the estimated and true number of completed reps at 2RIR and 4RIR were documented and further analyzed.

### Statistical analysis

Data were recorded using Microsoft Excel (Version 16.103.2) and analyzed using R (version 4.1.1; The R Foundation for Statistical Computing) within RStudio (version 2024.12.1 + 563, Posit Software, PBC). RIR estimation accuracy was quantified using both the raw directional difference (Δ), defined as the mean difference between true and estimated reps, and the mean absolute error (MAE), defined as the mean of the absolute differences between true and estimated reps. Both outcomes were averaged across the three sets and calculated separately for 2RIR and 4RIR for each of the six experimental sessions. Set-level differences were examined prior to averaging using separated mixed-effects models and are reported in Supplementary Figure S1 and Supplementary Table S1.

Baseline differences between groups were assessed using Welch’s independent-sample *t*-tests to account for potential heterogeneity of variances. To formally test the exercise- and RIR-target-specific patterns in estimation accuracy, paired t-tests were conducted on participant-level mean MAE, averaged across sessions and, for each comparison, across the respective other factor. Normality of the paired differences was assessed using the Shapiro–Wilk test. For descriptive purposes, associations between total reps to failure and MAE were examined using Pearson correlation coefficients (*r*), calculated separately for each exercise and RIR condition, pooled across age groups. Correlation coefficients were interpreted using benchmarks (negligible ≈ 0.00–0.09, weak ≈ 0.10–0.39, moderate ≈ 0.40–0.69, strong ≈ 0.70–0.89, very strong ≈ 0.90–1.00) commonly applied to *r* [21]. Longitudinal changes in MAE were examined using linear mixed-effects models (LMMs), fitted separately for each exercise (bench press and leg press) and separately for 2RIR and 4RIR. Each model included session, group, and their interaction as fixed effects, with random intercepts for participants to account for repeated measurements across sessions. Effect estimates are reported as fixed-effect coefficients with 95% confidence intervals (95%-CI), reflecting change in MAE associated with each model term.

As a complementary summary, Type III *F*-tests with Satterthwaite-approximated denominator degrees of freedom, as implemented in the lmerTest package, and partial eta squared (*η*^*2*^*ₚ*) with corresponding 95%-CI (effectsize package) are reported in the supplementary material. These F-tests, rather than the individual coefficient p-values, serve as the primary test of statistical significance for each fixed effect, since coefficient-level estimates are reported descriptively and without adjustment for multiple comparisons.

The four drop-out participants were excluded from the final analyses. Among participants who completed the study, no outcome data were missing; therefore, no imputation or other missing-data procedures were applied. Unless stated otherwise, data are presented as group means (M) ± standard deviation (SD) or as M [95%-CI].

## Results

Participants’ baseline characteristics are presented in Table 1*.* At baseline, apart from age, only LP 1RM differed significantly between groups (*t*(21.03) = −2.91, *p* = 0.008). Across all sessions, average reps per set were 10.9 ± 1.8 (BP) and 13.3 ± 3.0 (LP) for younger adults, and 11.0 ± 2.3 (BP) and 14.6 ± 4.1 (LP) for older adults. Both groups underestimated the number of reps at 2RIR and 4RIR for both BP and LP.

**Table 1:** Baseline characteristics of participants

	Younger adults		Older adults	
	M ± SD	Range	M ± SD	Range
*n (f, m)*	*13 (9, 4)*		*13 (7, 6)*	
Age [years]	33 ± 7	19—40	67 ± 4	60—74
Height [cm]	173 ± 7	163—186	173 ± 7	160—183
Body mass [kg]	74 ± 12	54—92	77 ± 12	62—110
1RM BP [kg]	37.5 ± 15.0	26.0—75.0	29.5 ± 13.0	16—57.5
1RM LP [kg]	132 ± 15	96—168	96 ± 37	48—170

*M* Mean, *SD* Standard deviation, *n* sample size, *f* female, *m* male, *1RM* one repetition maximum, *BP* Bench press, *LP* Leg press

Paired t-tests confirmed that MAE was significantly lower for BP than LP (mean difference = −1.42 reps, 95%-CI [−1.96, −0.88], t(25) = −5.44, *p* < 0.001) and significantly lower at 2RIR than 4RIR (mean difference = −1.04 reps, 95%-CI [−1.30, −0.78], t(25) = −8.14, *p* < 0.001).

Across both age groups, significant (*p* < 0.01) descriptive correlations were observed between total reps to failure and MAE. Correlations were consistently stronger for 4RIR than for 2RIR. Correlations were moderate for LP at 2RIR (*r* ≈ 0.42–0.50) and 4RIR (*r* ≈ 0.54–0.58) and weak for BP at 2RIR (*r* ≈ 0.19–0.26) but moderate at 4RIR (*r* ≈ 0.47–0.55).

**Fig. 1:**
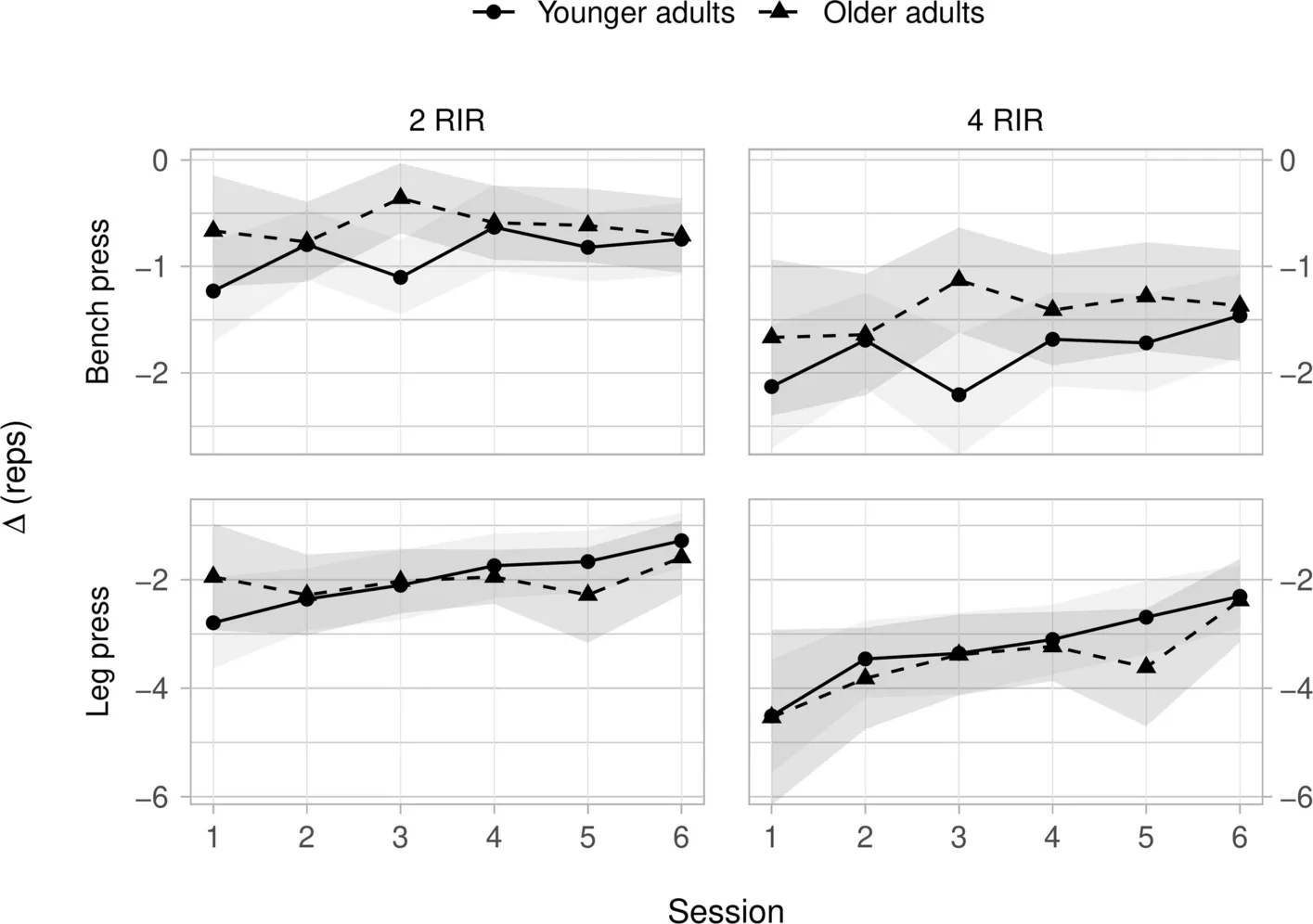
Time course of Δ (reps) across sessions for 2RIR and 4RIR. The colored areas represent the 95% confidence intervals. 2RIR, two repetitions in reserve; Δ, directional mean error; reps, repetitions

**Fig. 2:**
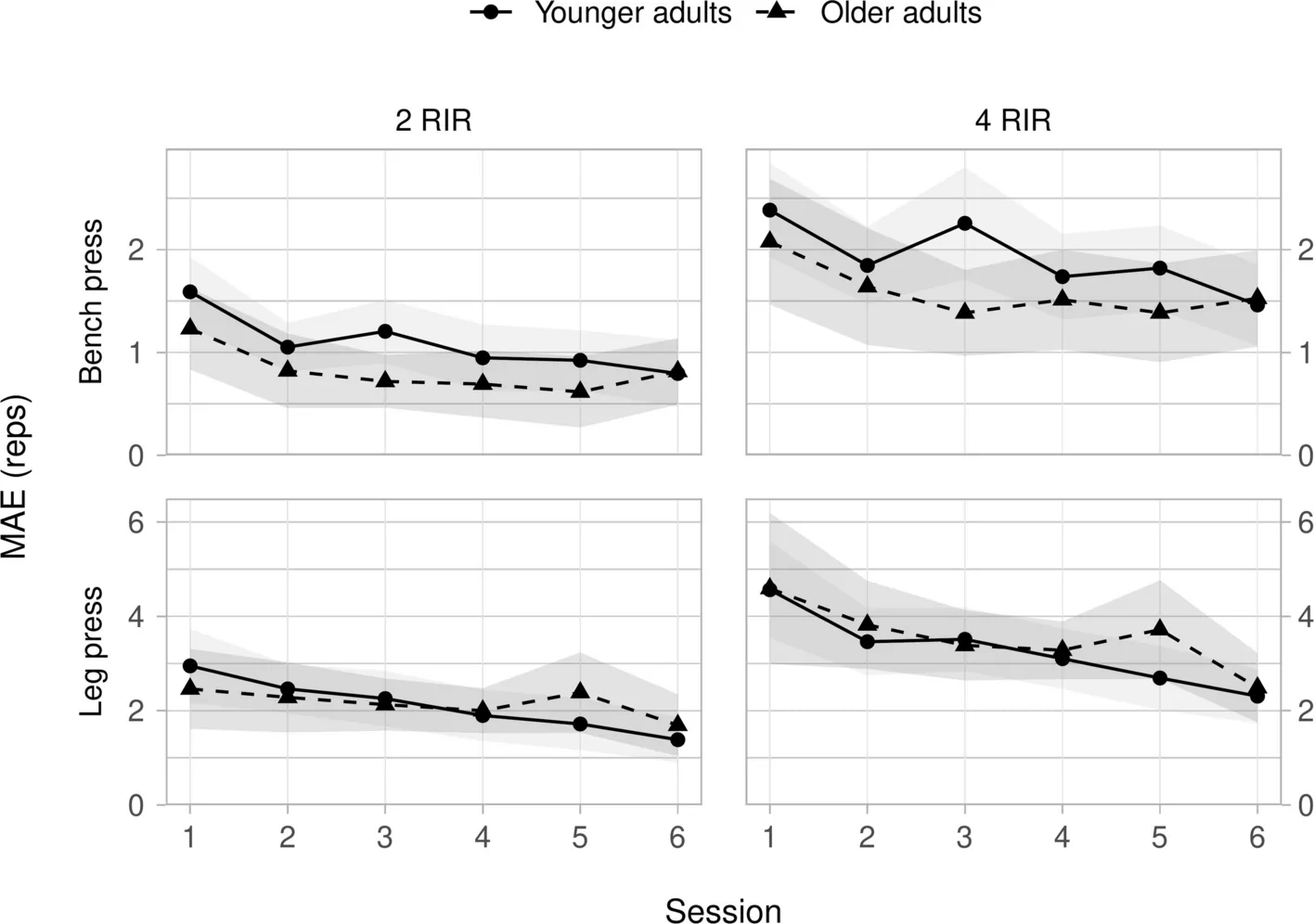
Time course of MAE (reps) across sessions for 2RIR and 4RIR. The colored areas represent the 95% confidence intervals. 2RIR, two repetitions in reserve; MAE, mean absolute error; reps, repetitions

Differences in Δ and MAE across sessions are illustrated in Figs. 1 and 2, with corresponding values provided in Supplementary Tables S2 and S3. In younger adults, MAE decreased by 0.8 reps for BP (Session 1 = 1.6 [1.1,2.1]; Session 6 = 0.8 [0.4,1.1]) and 1.5 reps for LP (Session 1 = 2.9 [2.0,3.9]; Session 6 = 1.4 [0.6,2.1]) at 2RIR. At 4RIR, MAE was reduced by 0.9 reps for BP (Session 1 = 2.4 [1.7,3.1]; Session 6 = 1.5 [1.0,2.0]) and 2.3 reps for LP (Session 1 = 4.6 [3.3,5.8]; Session 6 = 2.3 [1.6,3.0]).

Similarly, in older adults MAE decreased by 0.4 reps for BP (Session 1 = 1.2[0.6,1.8]; Session 6 = 0.8 [0.2,1.4]) and 0.8 reps for LP (Session 1 = 2.5 [1.4 ,3.5]; Session 6 = 1.7 [0.7,2.7]) at 2RIR, and by 0.7 reps for BP (Session 1 = 2.1 [1.0,3.1]; Session 6 = 1.5 [0.7,2.3]) and 2.1 reps for LP (Session 1 = 4.6 [2.0,7.2]; Session 6 = 2.5 [1.5,3.5]) at 4RIR.

LMM coefficients (Table 2) indicated significantly lower MAE than Session 1 from Session 4 onward across all four models (and additionally at Session 2 for BP at 2RIR), consistent with significant *F*-tests for the main effect of session for both BP and LP (*p* < 0.01; Supplementary Table S4). No significant group differences were observed at any session (Table 2), and the *F*-test evaluating the five session x group interaction terms was non-significant for all four models (Supplementary Table S4). Intercepts and session x group interaction coefficients are reported in Supplementary Table S5.

**Table 2:** Fixed-effect coefficients from linear mixed-effects models of MAE at 2RIR and 4RIR in bench press and leg press

	2RIR			4RIR		
	Estimate [reps]	95%-CI	*p*	Estimate [reps]	95%-CI	*p*
a) Bench press						
Session 2 vs. 1	−0.54	−0.99 – −0.09	0.019	−0.54	−1.17 – 0.09	0.092
Session 3 vs. 1	−0.38	−0.83 – 0.07	0.093	−0.13	−0.76 – 0.5	0.687
Session 4 vs. 1	−0.65	−1.1 – −0.2	0.005	−0.67	−1.29 – −0.04	0.037
Session 5 vs. 1	−0.67	−1.12 – −0.22	0.004	−0.56	−1.19 – 0.06	0.078
Session 6 vs. 1	−0.79	−1.24 – −0.35	<.001	−0.92	−1.55 – −0.3	0.004
Group (Older vs. Younger)	−0.36	−0.96 – 0.24	0.234	−0.31	−1.26 – 0.65	0.522
b) Leg press						
Session 2 vs. 1	−0.49	−1.29 – 0.32	0.233	−1.1	−2.25 – 0.05	0.060
Session 3 vs. 1	−0.69	−1.5 – 0.11	0.091	−1.05	−2.2 – 0.1	0.072
Session 4 vs. 1	−1.05	−1.86 – −0.25	0.011	−1.46	−2.61 – −0.31	0.013
Session 5 vs. 1	−1.23	−2.04 – −0.43	0.003	−1.87	−3.02 – −0.72	0.002
Session 6 vs. 1	−1.56	−2.37 – −0.76	<.001	−2.26	−3.4 – −1.11	<.001
Group (Older vs. Younger)	−0.49	−1.72 – 0.74	0.430	0.03	−1.71 – 1.76	0.976

*MAE* Mean absolute error, *2RIR* two repetitions in reserve, *4RIR* four repetitions in reserve, *reps* repetitions, *CI* Confidence interval, *p* p-values (Satterthwaite-approximated df)

## Discussion

To the best of our knowledge, the present study is the first to investigate the accuracy of RIR estimation and its trainability in older adults across multiple training sessions. The main findings indicate that RIR estimation accuracy improved systematically over time for both age groups, with no evidence of age-related statistical differences in trainability. Notably, estimation accuracy appeared to be context specific. It displayed a weak to moderate correlation with the total number of reps until muscle failure, was consistently higher at two reps in reserve (2RIR) compared with four reps in reserve (4RIR) and higher for bench press (BP) than for leg press (LP). The systematic improvement in estimation accuracy over time supports the presence of a training effect. Thereby, this finding adds valuable insight to the previously equivocal body of evidence [13–16, 18] on the trainability of RIR estimation accuracy.

The inconsistent findings reported across previous studies may, at least in part, be attributed to the heterogeneity in study designs, investigated populations and other intervention characteristics. In the present study, participants generally exhibited the lowest accuracy in Session 1 and, apart from BP in older adults, the highest accuracy in Session 6. The observed training effect became evident after the first training session and progressively consolidated until Session 6. Although participants received no explicit feedback on their estimation accuracy, completing every set to true concentric muscle failure inherently provided intrinsic, self-generated information about estimation error, which may have contributed to the observed improvement across sessions [22]. This form of intrinsic feedback, arising naturally from exercise performance rather than external correction, may be sufficient to improve self-monitoring accuracy over repeated exposures. Increasing comfort and confidence in approaching true concentric muscle failure within a supervised setting may also have contributed.

It is unclear whether some of the participants may have improved even further with additional training sessions beyond the study period. Previous evidence remains inconclusive regarding the influence of intervention duration on improvements in RIR estimation, with mixed findings reported for both, multiple weeks of training [14, 16] and only a single training session [13, 15]. These inconsistencies suggest that factors beyond intervention duration warrant consideration when interpreting the difference in RIR estimation trainability across studies. Notably, previous studies have predominantly examined resistance-trained individuals with multiple years of systematic resistance training experience [13, 14, 16], and in one case even participants with a competitive background in strength sports and experience with RIR-based training [13]. Interestingly, the two studies reporting improvements in RIR estimation accuracy following training included participants with a less advanced training status [15] or no prior RIR estimation experience [16], which more closely resembles the population examined in this present study. Novice individuals with little to no prior training experience typically show disproportionately large early RT improvements compared with trained individuals [23]. Although participants in the present study were not necessarily novices to resistance training itself, they had no prior experience specifically with RIR-based self-monitoring. Thus, a similar pattern of pronounced early-stage improvement in individuals new to a given skill may partly explain the gains in RIR estimation accuracy observed here, irrespective of any shared underlying mechanisms with strength adaptations. As both younger and older participants had comparably limited RIR self-monitoring experience, this pattern would be expected to apply similarly across age groups, consistent with the absence of significant group differences in trainability observed in the present study. Direct experimental investigations examining training status and familiarity with RIR-based training as a confounding factor for RIR estimation accuracy and its trainability are currently lacking, with no conclusive evidence demonstrating an independent effect of training status on estimation accuracy at baseline [9, 10]. However, it is apparent that the two studies which did not observe a meaningful training effect [13, 14] displayed already lower mean absolute estimation errors at baseline (≲ 1 rep) than the present study and previous investigations which have indicated a trainability [15, 16]. These observations appear consistent with a potential ceiling effect, whereby low baseline errors, in already well-trained individuals, may limit the magnitude of further improvement. It is plausible that mean estimation accuracy will not substantially improve beyond a certain point.

Furthermore, the findings of the present study not only support the trainability of RIR estimation accuracy but also provide novel insight into the general accuracy for RIR estimation in older adults. Specifically, the present results indicate comparable baseline estimation accuracy for younger and older adults. The only previous study investigating RIR estimation accuracy in older adults reported directional errors (not MAE) of −1.6 ± 0.6 and −2.1 ± 0.3 reps for 4RIR and 2RIR, respectively, during a chest press exercise, corresponding to estimated 95% CIs of [−1.85, −1.35] and [−2.22, −1.98] (estimated from reported mean ± SD, *n* = 25) [18]. For reference, in the current study BP 4RIR estimates overlapped with this value at both Session 1 (Δ = −1.7 [95% CI −2.8, −0.5]) and Session 6 (Δ = −1.3 [−2.2, −0.4]), whereas our 2RIR estimates did not, at either Session 1 (Δ = −0.7 [−1.5, 0.1]) or Session 6 (Δ = −0.7 [−1.3, −0.1]) (Supplementary Table S2). However, Gómez-Redondo and colleagues did not assess true estimation accuracy within the same training session but instead assessed reps to failure for the target load in a separate session from the actual RIR estimation task, during which participants stopped their set based on their estimated RIR. This design choice may have introduced considerable error variance through intraindividual day-to-day variability in performance. This consideration is particularly relevant when investigating older adults, who have been demonstrated to exhibit greater inter- and intraindividual variability in RT performance [12]. It may also have allowed discomfort- or injury-related caution to influence the voluntary stopping point specifically when closer to failure, as the original authors themselves suggest. This is consistent with the divergence being concentrated at 2RIR rather than 4RIR.

Consistent with previous evidence, the present study demonstrated larger estimation errors at 4RIR compared with 2RIR [9, 10]. This finding further supports the well-established notion that RIR estimation accuracy increases with greater proximity to concentric muscle failure and suggests that this relationship also applies to older adults, a population that has received limited attention in prior research [9–11]. As previously discussed, this pattern may be explained by the increased perceptibility of fatigue-related cues and the reduced range of possible remaining reps as sets approach concentric muscle failure, which may facilitate more accurate self-monitoring [9–11].

The present study demonstrates that RIR estimation accuracy was superior in BP compared with LP. This is in line with several previous studies observing accuracy differences between upper and lower body exercises [15, 24–26]. However these initial findings do not hold up in meta-analysis and appear to be uncertain [10].

Interestingly, the apparent differences in RIR estimation accuracy may be better explained by the total number of reps performed until concentric muscle failure within each set. In the present investigation, participants completed, on average, approximately three more reps during the LP compared with the BP. As highlighted in previous reviews and meta-analyses, estimation error appears to increase alongside the total reps performed until concentric muscle failure within a set [9–11]. Similar to the effect of proximity to failure, this pattern may reflect the increased perceptibility of fatigue-related cues in shorter sets, as well as the mathematically reduced range of possible reps, both of which may facilitate more accurate RIR estimation [9, 11]. These findings, supported by previous evidence, suggest that RIR prescription should be guided by scenario specific requirements.

In most RT scenarios, RIR targets may be most valuable as a tool for preventing insufficient training effort. While training to true concentric muscular failure is likely not always necessary or advisable for older adults, they tend to train more conservatively [1, 18] than evidence would support [27, 28]. High-load and high-effort RT has been shown to be feasible and effective in older adults [27, 29, 30]. Thus, RIR-targets closer to concentric muscle failure (e.g., 2RIR) may be preferable for RT prescription in general, and particularly in scenarios requiring a tighter control of RT effort, such as research settings. Sets with larger RIR targets (e.g., 4RIR) are, inherently, stopped considerably before the set’s concentric muscle failure. Thus, while an estimation error of less than three reps is unlikely to meaningfully influence overall training stress, it may still blunt the desired training outcome by not reaching an already conservative target [5]. RT sets performed with lower loads and higher reps, as well as RIR targets further from concentric muscle failure, therefore likely require more familiarization than provided in the present study.

Although the present study provides novel insights into RIR estimation accuracy and its trainability in older adults, some limitations should be acknowledged. The a-priori sample size calculation targeted the detection of time-related (session) effects, the primary aim of the present study, and the required sample size of 24 participants was exceeded. However, the non-significant findings for group and session x group effects should not be interpreted as evidence of true equivalence between age groups, as the present study was not designed to formally test statistical equivalence, which would require a dedicated equivalence-testing approach and a correspondingly calculated sample size. Additionally, because the primary analysis relied on LMMs rather than a repeated-measures ANOVA, the a-priori power calculation based on ANOVA-specific assumptions, does not translate exactly onto the random-effects structure of the LMM. While both approaches address the same repeated-measures design and are expected to yield broadly comparable power for the primary session effect, this mismatch between the analytic frameworks used for planning and analysis should be acknowledged as a limitation of the a-priori sample size estimation. Another limitation concerns the interpretability of the RIR estimation error, which is measured in whole repetitions. Group-level MAE reductions of less than one rep therefore do not necessarily indicate a meaningful change for any given individual. Furthermore, exercise order was not counterbalanced across sessions, with every session beginning with LP followed by BP. This constitutes a design limitation, as fixed-order effects on the comparison of LP and BP cannot be entirely ruled out. However, the order was constant across sessions and both age groups, and thus unlikely to have influenced the main outcomes of this study. A further limitation is that the six training sessions were spaced over six weeks. Other training frequencies (e.g., multiple sessions per week) may yield different outcomes and a distinct time course. Therefore, our findings should not be generalized beyond the tested schedule. Further research is therefore warranted to strengthen the evidence regarding RIR estimation in older adults. Future studies should also directly examine participants’ training status as a potential moderator of RIR estimation trainability and explore the time course of training effects beyond six weeks to determine whether improvements in estimation accuracy may reach a ceiling after a certain training duration.

## Conclusion

In conclusion, RIR estimation accuracy improved systematically across six consecutive training sessions for both BP and LP in younger and older adults, with no evidence of age-related differences in baseline accuracy or trainability. These findings indicate that RIR-based resistance training approaches are feasible in older adults, provided a brief familiarization period of four to six sessions over multiple weeks is ensured. This may be particularly relevant for exercise prescription in aging populations, where greater intra- and interindividual variability in training tolerance increases the need for individualized training stimuli, and where insufficient RT effort and intensity may be a concern. In most applied training settings, RIR targets closer to concentric muscle failure (e.g., 2RIR) may be preferable because absolute estimation errors of one to two reps per set are unlikely to meaningfully affect training adaptations. In scenarios where more precise effort control is desired, such as research settings, exercises or loading schemes characterized by fewer total reps to failure may facilitate more accurate RIR estimation.

## Supplementary Information

Supplementary Material 1.

## Data Availability

The datasets analyzed during the current study are openly available in the zenodo repository at: 10.5281/zenodo.18506148

## Abbreviations

RT: Resistance training
RIR: Repetitions in reserve
reps: Repetitions
1RM: One-repetition maximum
BP: Bench press
LP: Leg press
MAE: Mean absolute error
Δ: Directional mean error
LMM: Linear mixed-effects model
CI: Confidence interval
SD: Standard deviation
M: Mean
n: Sample size
f: Female
m: Male
η^2^ₚ: Partial eta squared
RPE: Rating of perceived exertion
