# Supplementary material for: Trainability of repetitions-in-reserve estimation in younger and older adults: a parallel-group repeated-measures study
Source: BMC Sports Sci Med Rehabil. 2026 Aug 20;18:375. doi: 10.1186/s13102-026-01997-y (PMC13499321; doi:10.1186/s13102-026-01997-y)
Supplement: Supplementary file 1 [file 13102_2026_1997_MOESM1_ESM.docx]

***Supplementary Figure S1:*** *Set-level MAE (reps) across the three sets for bench press and leg press at 2RIR and 4RIR, shown separately for younger and older adults. The colored areas represent the 95% confidence intervals. 2RIR, two repetitions in reserve; 4RIR, four repetitions in reserve; MAE, mean absolute error; reps, repetitions*

**Supplementary Table S1:** Bonferroni-adjusted pairwise comparisons of mean absolute error between sets at 2RIR and 4RIR in bench press and leg press

| **Outcome** | **Contrast** | **Estimate (reps)** | **SE** | **df** | **t** | **p (Bonferroni)** |
| --- | --- | --- | --- | --- | --- | --- |
| **a) Bench press** | |  |  |  |  |  |
| 2 RIR | Set 1 – Set 3 | 0.330 | 0.102 | 445 | 3.23 | **0.004** |
|  | Set 2 – Set 3 | 0.326 | 0.102 | 445 | 3.19 | **0.005** |
| 4 RIR | Set 1 – Set 2 | -0.386 | 0.136 | 445 | -2.85 | **0.014** |
|  | Set 2 – Set 3 | 0.494 | 0.136 | 445 | 3.64 | **0.001** |
| **b) Leg press** | |  |  |  |  |  |
| 2 RIR | Set 1 – Set 2 | 0.500 | 0.185 | 446 | 2.70 | **0.022** |
|  | Set 1 – Set 3 | 0.753 | 0.185 | 446 | 4.06 | **<0.001** |
| 4 RIR | Set 1 – Set 3 | 0.789 | 0.249 | 447 | 3.17 | **0.005** |

*Note: Estimates are differences in estimated marginal means derived from separate linear mixed-effects models fitted on set-level MAE for each exercise and RIR target, with set number (Set 1 – Set 3) and age group as fixed factors and a random intercept for participant. Comparisons are averaged over age groups; degrees of freedom were Satterthwaite-approximated, and p-values and confidence intervals are Bonferroni-adjusted for the three pairwise comparisons per model. All pairwise comparisons are reported. MAE, mean absolute error; 2RIR, two repetitions in reserve; 4RIR, four repetitions in reserve; reps, repetitions; 95%-CI, 95% confidence interval.*

**Supplementary Table S2:** Directional mean error of repetitions with standard deviation and 95% confidence interval for each session.

|  | **Younger adults** | | | | | | **Older adults** | | | | | |
| --- | --- | --- | --- | --- | --- | --- | --- | --- | --- | --- | --- | --- |
|  | 2RIR | | | 4RIR | | | 2RIR | | | 4RIR | | |
|  | *∆* | *SD* | *95%-CI* | *∆* | *SD* | *95%-CI* | *∆* | *SD* | *95%-CI* | *∆* | *SD* | *95%-CI* |
| **a) Bench press** | |  |  |  |  |  |  |  |  |  |  |  |
| **1** | -1.2 | 1.1 | **-1.9 – -0.5** | -2.1 | 1.5 | **-3.0 – -1.2** | -0.7 | 1.3 | **-1.5 – 0.1** | -1.7 | 2.0 | **-2.8 – -0.5** |
| **2** | -0.8 | 0.8 | **-1.2 – -0.3** | -1.7 | 1.1 | **-2.4 – -1.0** | -0.8 | 0.9 | **-1.3 – -0.2** | -1.6 | 1.4 | **-2.5 – -0.8** |
| **3** | -1.1 | 0.8 | **-1.6 – -0.6** | -2.2 | 1.3 | **-3.0 – -1.4** | -0.4 | 0.8 | **-0.8 – 0.1** | -1.1 | 1.4 | **-1.9 – -0.3** |
| **4** | -0.6 | 1.0 | **-1.2 – -0.0** | -1.7 | 1.1 | **-2.3 – -1.0** | -0.6 | 0.8 | **-1.1 – -0.1** | -1.4 | 1.3 | **-2.2 – -0.6** |
| **5** | -0.8 | 0.7 | **-1.2 – -0.4** | -1.7 | 1.0 | **-2.3 – -1.1** | -0.6 | 0.9 | **-1.1 – -0.1** | -1.3 | 1.1 | **-1.9 – -0.6** |
| **6** | -0.7 | 0.7 | **-1.1 – -0.3** | -1.5 | 0.8 | **-2.0 – -1.0** | -0.7 | 1.0 | **-1.3 – -0.1** | -1.3 | 1.5 | **-2.2 – -0.4** |
| **b) Leg press** | |  |  |  |  |  |  |  |  |  |  |  |
| **1** | -2.8 | 1.6 | **-3.8 – -1.8** | -4.5 | 2.2 | **-5.8 – -3.2** | -1.9 | 2.3 | **-3.3 – -0.6** | -4.5 | 4.3 | **-7.2 – -1.9** |
| **2** | -2.4 | 1.2 | **-3.1 – -1.6** | -3.5 | 1.3 | **-4.3 – -2.6** | -2.3 | 2.1 | **-3.6 – -1.0** | -3.8 | 2.7 | **-5.4 – -2.2** |
| **3** | -2.1 | 1.3 | **-2.9 – -1.3** | -3.4 | 1.5 | **-4.2 – -2.5** | -2.0 | 1.7 | **-3.0 – -1.0** | -3.4 | 2.1 | **-4.6 – -2.1** |
| **4** | -1.7 | 1.3 | **-2.5 – -1.0** | -3.1 | 1.4 | **-3.9 – -2.3** | -1.9 | 1.4 | **-2.8 – -1.1** | -3.2 | 1.8 | **-4.3 – -2.2** |
| **5** | -1.7 | 1.2 | **-2.4 – -0.9** | -2.7 | 1.5 | **-3.6 – -1.8** | -2.3 | 2.5 | **-3.8 – -0.8** | -3.6 | 3.1 | **-5.5 – -1.7** |
| **6** | -1.3 | 1.3 | **-2.1 – -0.5** | -2.3 | 1.2 | **-3.0 – -1.6** | -1.6 | 1.5 | **-2.5 – -0.7** | -2.4 | 1.6 | **-3.3 – -1.4** |

2RIR, *two repetitions in reserve*; 4RIR, *four repetitions in reserve*; MAE, *mean absolute error*; SD, *standard deviation*; 95%-CI, *95% confidence interval*

**Supplementary Table S3:** Mean absolute error of repetitions with standard deviation and 95% confidence interval for each session.

|  | **Younger adults** | | | | | | **Older adults** | | | | | |
| --- | --- | --- | --- | --- | --- | --- | --- | --- | --- | --- | --- | --- |
|  | 2RIR | | | 4RIR | | | 2RIR | | | 4RIR | | |
|  | *MAE* | *SD* | *95%-CI* | *MAE* | *SD* | *95%-CI* | *MAE* | *SD* | *95%-CI* | *MAE* | *SD* | *95%-CI* |
| **a) Bench press** | |  |  |  |  |  |  |  |  |  |  |  |
| **1** | 1.6 | 0.8 | **1.1 – 2.1** | 2.4 | 1.1 | **1.7 – 3.1** | 1.2 | 1.0 | **0.6 – 1.8** | 2.1 | 1.7 | **1.0 – 3.1** |
| **2** | 1.1 | 0.5 | **0.7 – 1.4** | 1.8 | 0.9 | **1.3 – 2.4** | 0.8 | 0.9 | **0.3 – 1.4** | 1.6 | 1.4 | **0.8 – 2.5** |
| **3** | 1.2 | 0.7 | **0.8 – 1.6** | 2.3 | 1.3 | **1.5 – 3.1** | 0.7 | 0.6 | **0.4 – 1.1** | 1.4 | 1.2 | **0.7 – 2.1** |
| **4** | 0.9 | 0.7 | **0.5 – 1.4** | 1.7 | 1.0 | **1.1 – 2.3** | 0.7 | 0.8 | **0.2 – 1.2** | 1.5 | 1.3 | **0.8 – 2.3** |
| **5** | 0.9 | 0.6 | **0.6 – 1.3** | 1.8 | 0.9 | **1.3 – 2.4** | 0.6 | 0.9 | **0.1 – 1.1** | 1.4 | 1.3 | **0.6 – 2.2** |
| **6** | 0.8 | 0.6 | **0.4 – 1.1** | 1.5 | 0.8 | **1.0 – 2.0** | 0.8 | 0.9 | **0.2 – 1.4** | 1.5 | 1.4 | **0.7 – 2.3** |
| **b) Leg press** | |  |  |  |  |  |  |  |  |  |  |  |
| **1** | 2.9 | 1.5 | **2.0 – 3.9** | 4.6 | 2.1 | **3.3 – 5.8** | 2.5 | 1.7 | **1.4 – 3.5** | 4.6 | 4.3 | **2.0 – 7.2** |
| **2** | 2.5 | 1.1 | **1.8 – 3.2** | 3.5 | 1.3 | **2.6 – 4.3** | 2.3 | 2.1 | **1.0 – 3.6** | 3.8 | 2.7 | **2.2 – 5.4** |
| **3** | 2.3 | 1.2 | **1.6 – 3.0** | 3.5 | 1.4 | **2.7 – 4.3** | 2.1 | 1.5 | **1.2 – 3.1** | 3.4 | 2.1 | **2.1 – 4.6** |
| **4** | 1.9 | 1.3 | **1.1 – 2.7** | 3.1 | 1.4 | **2.3 – 3.9** | 2.0 | 1.3 | **1.2 – 2.8** | 3.3 | 1.7 | **2.3 – 4.3** |
| **5** | 1.7 | 1.1 | **1.0 – 2.4** | 2.7 | 1.5 | **1.8 – 3.6** | 2.4 | 2.4 | **0.9 – 3.8** | 3.7 | 3.1 | **1.9 – 5.6** |
| **6** | 1.4 | 1.2 | **0.6 – 2.1** | 2.3 | 1.2 | **1.6 – 3.0** | 1.7 | 1.6 | **0.7 – 2.7** | 2.5 | 1.7 | **1.5 – 3.5** |

2RIR, *two repetitions in reserve*; 4RIR, *four repetitions in reserve*; MAE, *mean absolute error*; SD, *standard deviation*; 95%-CI, *95% confidence interval*

**Supplementary Table S4:** Results of linear mixed-effects models for MAE at 2RIR and 4RIR in bench press and leg press

| **Outcome** | | **Effect** | **df1** | **df2** | **F** | ***p*** | **η²ₚ** |
| --- | --- | --- | --- | --- | --- | --- | --- |
| **a) Bench press** | | |  |  |  |  |  |
| MAE (2RIR) | Session | | 5 | 120 | 4.42 | .001 | 0.16 |
|  | Group | | 1 | 24 | 1.55 | .225 | 0.06 |
|  | Session × Group | | 5 | 120 | 0.54 | .748 | 0.02 |
| MAE (4RIR) | Session | | 5 | 120 | 2.75 | .022 | 0.10 |
|  | Group | | 1 | 24 | 0.75 | .395 | 0.03 |
|  | Session × Group | | 5 | 120 | 0.96 | .445 | 0.04 |
| **b) Leg press** | | |  |  |  |  |  |
| MAE (2RIR) | Session | | 5 | 120 | 3.78 | .003 | 0.14 |
|  | Group | | 1 | 24 | 0.01 | .924 | 0.00 |
|  | Session × Group | | 5 | 120 | 0.99 | .424 | 0.04 |
| MAE (4RIR) | Session | | 5 | 120 | 6.02 | < .001 | 0.20 |
|  | Group | | 1 | 24 | 0.16 | .693 | 0.01 |
|  | Session × Group | | 5 | 120 | 0.48 | .788 | 0.02 |

Note: 95%-CI, *95% confidence interval*; 2RIR, *two repetitions in reserve*; 4RIR, *four repetitions in reserve*; MAE, *mean absolute error*; SD, *standard deviation*

**Supplementary Table S5:** Additional fixed-effect coefficients (Intercept and Session x Group interaction) from linear mixed-effects models of MAE at 2RIR and 4RIR in bench press and leg press

|  | **2RIR** | | | **4RIR** | | |
| --- | --- | --- | --- | --- | --- | --- |
|  | **Estimate [reps]** | **95%-CI** | **p** | **Estimate [reps]** | **95%-CI** | **p** |
| **a) Bench press** |  |  |  |  |  |  |
| Intercept (Session 1, Younger adults) | 1.59 | 1.17 – 2.01 | < .001 | 2.38 | 1.71 – 3.06 | < .001 |
| Session 2 vs. 1 x Group | 0.13 | -0.51 – 0.76 | 0.691 | 0.10 | -0.78 – 0.99 | 0.819 |
| Session 3 vs. 1 x Group | -0.13 | -0.76 – 0.51 | 0.691 | -0.56 | -1.45 – 0.32 | 0.211 |
| Session 4 vs. 1 x Group | 0.12 | -0.52 – 0.75 | 0.720 | 0.10 | -0.78 – 0.99 | 0.819 |
| Session 5 vs. 1 x Group | 0.05 | -0.58 – 0.69 | 0.873 | -0.13 | -1.02 – 0.76 | 0.775 |
| Session 6 vs. 1 x Group | 0.37 | -0.26 – 1.01 | 0.250 | 0.36 | -0.53 – 1.25 | 0.425 |
| **b) Leg press** |  |  |  |  |  |  |
| Intercept (Session 1, Younger adults) | 2.95 | 2.08 – 3.82 | < .001 | 4.56 | 3.34 – 5.79 | < .001 |
| Session 2 vs. 1 x Group | 0.31 | -0.83 – 1.45 | 0.594 | 0.33 | -1.29 – 1.96 | 0.685 |
| Session 3 vs. 1 x Group | 0.36 | -0.78 – 1.5 | 0.534 | -0.15 | -1.78 – 1.47 | 0.852 |
| Session 4 vs. 1 x Group | 0,59 | -0.55 – 1.73 | 0.307 | 0.15 | -1.47 – 1.78 | 0.852 |
| Session 5 vs. 1 x Group | 1.15 | 0.02 – 2.29 | 0.047 | 1.00 | -0.62 – 2.62 | 0.225 |
| Session 6 vs. 1 x Group | 0.79 | -0.34 – 1.93 | 0.169 | 0.15 | -1.47 – 1.78 | 0.852 |

*Note: Given the non-significant F-test for the session x group interaction in this model (Supplementary Table S4), and given that 20 such interaction coefficients were tested across the four models, this isolated nominally significant term is consistent with a chance finding and should not be interpreted as evidence of a subgroup-specific effect.* MAE*, mean absolute error;* 2RIR*, two repetitions in reserve;* 4RIR*, four repetitions in reserve;* reps*, repetitions;* CI*, confidence interval.*
